# Response of Insect Relative Growth Rate to Temperature and Host-Plant Phenology: Estimation and Validation from Field Data

**DOI:** 10.1371/journal.pone.0086825

**Published:** 2014-01-22

**Authors:** Mamadou Ciss, Nicolas Parisey, Gwenaëlle Fournier, Pierre Taupin, Charles-Antoine Dedryver, Jean-Sébastien Pierre

**Affiliations:** 1 UMR 1349 IGEPP, Institut National de Recherche Agronomique, Le Rheu, France; 2 UMR CNRS 6553 ECOBIO, Université Rennes1, Rennes, France; 3 Service Génétique, Physiologie et Protection des Plantes, Arvalis-Institut-du-Végétal, Boigneville, France; Technion-Israel Institute of Technology Haifa, Israel

## Abstract

Between 1975 to 2011, aphid Relative Growth Rates (RGR) were modelled as a function of mean outdoor temperature and host plant phenology. The model was applied to the grain aphid *Sitobion avenae* using data on aphid counts in winter wheat at two different climate regions in France (oceanic climate, Rennes (western France); continental climate, Paris). Mean observed aphid RGR was higher in Paris compared to the Rennes region. RGR increased with mean temperature, which is explained by aphid reproduction, growth and development being dependent on ambient temperature. From the stem extension to the heading stage in wheat, there was either a plateau in RGR values (Rennes) or an increase with a maximum at heading (Paris) due to high intrinsic rates of increase in aphids and also to aphid immigration. From the wheat flowering to the ripening stage, RGR decreased in both regions due to the low intrinsic rate of increase in aphids and high emigration rate linked to reduced nutrient quality in maturing wheat. The model validation process showed that the fitted models have more predictive power in the Paris region than in the Rennes region.

## Introduction

In light of global climate change, more research is needed to study the effects of temperature trends and associated fluctuations on living organisms. Changes in populations of indigenous or invasive insect pests are one of the most important challenges facing ecology and crop protection [2]–[4]. Pest outbreaks must be modelled for forecasting and prospective purposes. Modelling insect population dynamics requires the incorporation of various driving factors. In particular, the vital stages in insect life cycles depend heavily and almost instantaneously on ambient temperature, the main abiotic factor influencing insect development.

Development of herbivorous insect species also depends on biotic driving factors such as the phenology of the host plant. Other biotic factors act at higher trophic levels on insect development and include predators, parasitoids and entomopathogens, which in turn depend on herbivore population accumulation to survive. The insect food web is temperature dependent because all organisms involved are ectothermal. The combined response of insects to two or more driving factors is particularly difficult to study experimentally because the interactions between these factors require a large experimental design and thus expensive testing chambers. Furthermore, *in vitro* results are often very different from what is observed in nature [5]–[7].

A field-based ecological study, although more arduous, is preferable to a laboratory-based one because it produces a realistic set of parameters. We, therefore, attempted to model the instantaneous increase of an insect population (Relative Growth Rate or RGR) from data obtained *in natura*.

To model insect RGRs, we used an approach based on several sets of data obtained from sampling winter wheat fields, with the goal of providing a robust and simple basis for further modelling and forecasting the populations of the grain aphid *Sitobion avenae*. This aphid is a major agricultural pest in Europe [8]–[10] causing direct damage in the spring by feeding on the sap of growing wheat and indirect ones by transmitting plant viruses [11], [12]. Two different regions were selected for field data collection: Rennes basin in western France, and the Paris basin; both having different agro-climatic characteristics likely to influence aphid biology. The Rennes region has an oceanic climate, with mild temperatures allowing parthenogenetic aphids and their natural enemies to survive on wheat during the winter. Conversely, in the more continental climate of the Paris region, parthenogenetic aphids generally do not overwinter *in situ* [13].

The basic premise behind our model is that the RGR of insects with overlapping generations, such as aphids, varies continuously. This variation is driven by environmental factors, among which temperature and host-plant phenology play a key role [1]. Predators, entomopathogens and parasitoids have a more variable role due to their complex interactions with both their prey and climate [14]. The influence of these predators can also be estimated provided that accurate sampling of these organisms is available [15]. These natural enemies were initially ignored in the model, for sake of simplicity, and then incorporated in samples for which data were available [16]. The RGR of the aphid population was then estimated for each sampling event, and related to temperature and plant growth stage; this relationship is non-linear [17]–[19].

Because RGR is a relative derivative, it is the logarithmic derivative of the population growth function; its estimation is obtained by smoothing splines. Although quite well known by statisticians [20]–[22], this method is very rarely used by ecologists, to such the extent that many classical textbooks do not even mention it [23]. Smoothing splines belong to the category of non-parametric estimators of an underlying deterministic function perturbed by environmental or endogenous noise. Empirical estimators based on first-order differences are very sensitive to weakly autocorrelated random noise whose extreme form is white noise, or Brownian motion. Stone, as early as 1985, showed that spline derivative estimators can achieve an optimal *L_2_* rate of convergence (in quadratic mean: 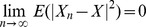 with *X_n_* is the spline derivative, *X* the convergence point and *n* is the number of samples).

In this study, we analysed field data from 1975 to 2011 in both Rennes and Paris regions in order to model *S. avenae* RGR linking to its driving factors. The prediction overall quality of the model was tested with two different datasets.

## Materials and Methods

### a) Sampling Methods

We collected the field data/samples ourselves and no permission is required to obtain them.

Aphid population densities were assessed from field counts: depending on winter wheat field infestation, fifty to 1000 tillers were randomly chosen from quadrats for observation each week from early May (stem extension stage) to mid-July (grain ripening stage) and numbers of living *S. avenae* per tiller were recorded. For each dataset, growth stages of wheat were recorded weekly according to Zadoks’ numerical scale [24]. For data collected in the Rennes region, numbers of *S. avenae* killed by entomopathogenic fungi (Entomophthorales) and insect parasitoid wasps (aphidiid ‘mummies’) were also recorded.

Minimum, maximum and mean temperature data were daily recorded in standard conditions (2 m high in a vented box) at a weather station near the fields (50 m to 1 km).

We worked on four different datasets, two for estimating the model parameters and two for validating the model. These latter two datasets were not used for fitting the model parameters, but only to check the predictive quality of the model. The first two datasets will hereafter be referred to as the *basic datasets*, and the latter two as the *test datasets*. Their content was as follows:

- the basic dataset for the Rennes region consists of *S. avenae* population densities recorded each year in a winter wheat field from 1975 to 1986 and in 1988, 1992, 1993, 2003 and 2004 (17 fields), at the INRA Research Centre at Le Rheu, (Ille et Vilaine *département*, France; 1°47′73′′W, 48°6′21′′N). The dataset includes 160 aphid and natural enemy counts on three successive wheat cultivars, Champlein (1975–1980), Arminda (1981–1993) and Lancelot (2003–2004) under variable weather conditions.
- the basic dataset for the Paris region consists of *S. avenae* population densities recorded in one wheat field for 12 years from 1980 to 2011, near the Arvalis research station in Boigneville (Essonne *département*, France; 48°20′06′′N, 2°22′14′′E). The dataset includes 102 aphid counts on successive wheat cultivars (Arminda, Thésée and Fidel being the most frequent), under variable weather conditions.
- the test dataset for the Rennes region consists of *S. avenae* population densities and the number of aphids killed by natural enemies, recorded in nine winter wheat fields from 1975 to 1982 for a total of 84 observations.
- the test dataset for the Paris region (47 observations) was recorded in 2004 in nine winter wheat fields selected in several areas around Paris.

### b) Statistical Analyses and Modelling Tools

For each field, the observed RGR at each sampling date was calculated from the grain aphid abundance curve. This curve was affected by strong noise resulting mainly from the sampling process. This noise made it difficult to estimate the derivatives with respect to time. Smoothing is known to improve greatly the estimates of the derivatives when the random fluctuations are faster than the underlying process [25]. Weekly data points were then smoothed by cubic splines [26]. A cubic spline is a function constructed from piecewise third-order polynomials that pass through a set of control points. For example, consider a collection of *n* known points 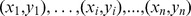. To obtain a smoothing spline for a given degree of smoothing (*i.e.* a given smoothing parameter), the control points are moved iteratively, using a technique such as the Gauss-Newton procedure, until a minimum in the residual sum of squares is reached (ordinary least squares). The choice of the smoothing parameter was carried out automatically by maximizing the cross-validation function (CVF, [25]), a feature included in the *smooth.spline* function in R [27]. To estimate the RGR of aphids, we considered the logarithmic derivative of the smoothing curve, which directly gave an estimate of the instantaneous relative rate of increase.

This instantaneous relative rate of increase (RGR) is well known in population dynamics [28] and the observed RGR was defined by:
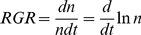
(1)where *n* is the number of aphids and *t* represents time (in days) and RGR’s unit measure is expressed as aphid/aphid/day (1/day).

This analysis of RGR was carried out in three steps:

1. Transformation of the time-series data on the number of aphids to *ln(n+1)* (to avoid the undefined logarithms for zero counts);
2. Smoothing of the logarithmic series using the cubic spline method; the smoothing parameter was chosen by finding the best value of the CVF [25]; the smoothed curve and the unsmoothed process were visually inspected to detect any anomalies. None occurred.
3. Calculation of the derivative of the spline functions at each observation time; this is the logarithmic derivative of the smoothed series and provides an estimate of RGR.

Although spline derivative estimators are generally robust, De Brabanter et al. [22] recently suggested a way to improve estimators by replacing the smoothing splines with a local polynomial regression, in an attempt to avoid choosing a smoothing parameter [29]. Nevertheless, in our case, the use of the cross-validation function (CVF) gave quite satisfying estimators growth and was therefore used to also estimate the derivatives.

### c) Modelling the Aphid RGR

We used non-linear regressions [30] of the weekly observed RGR calculated separately for each basic dataset on the weekly mean of mean daily temperatures and weekly wheat growth stage.

The non-linear regression model had the form:
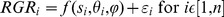
(2)where RGR*_i_* is the response variable, i.e. aphid RGR, *f* is a known function, *θ*_i_ the temperature, *s*_i_ the wheat growth stage, φ the parameter vector and ε_i_ random errors. The unknown parameter vector φ is estimated from the data by minimising the sum of the squared residuals.

Because the obtained data are likely to be autocorrelated within each field, a Durbin-Watson test was performed to evaluate this degree of autocorrelation. This test was used mainly as a precaution, because the main goal of this work was to estimate the parameters, not to test model significance.

We considered the overall quality of prediction for both basic and test datasets by considering the bisector (the line where the fitted values are equal to the observed values) and the residuals from the bisector considered as an ideal model. The ratio of the residual sum of squares over the overall sum of squares of the observed values, corrected by their degrees of freedom, is considered as an index of model quality, and subtracting this ratio from one is an index of goodness of fit. This index is equivalent to a coefficient of determination (R^2^). We set
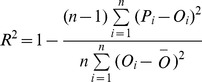
(3)where 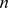 is the number of cases, 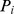 the *i* ^th^ predicted value, and 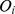 the *i* ^th^ observed value.

All calculations (non-linear regression, ANOVAs and Durbin-Watson tests) were carried out using R freeware [27].

## Results

### a) The Observed RGR

**Figure 1:**
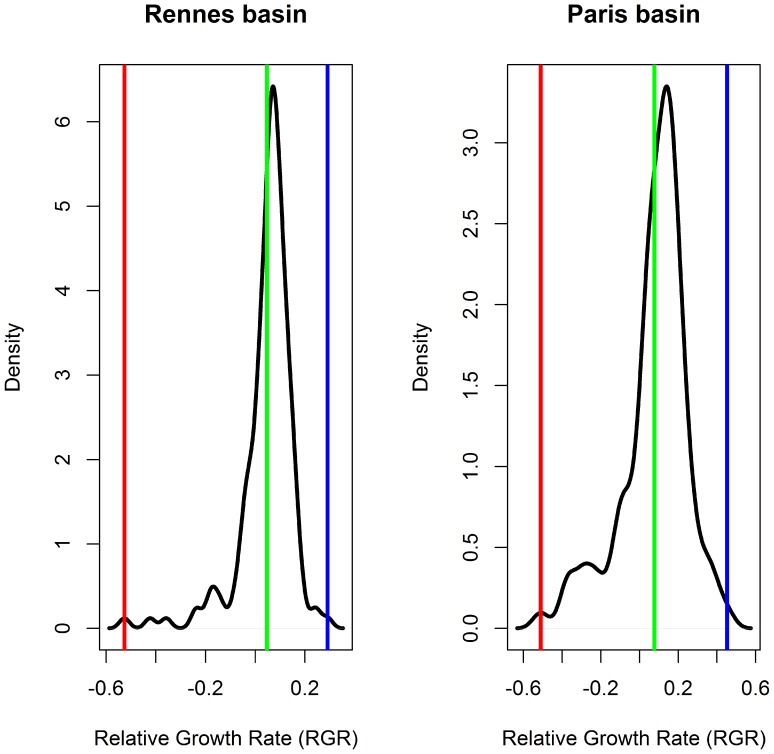
Comparison of observed relative growth rates for the Rennes basic dataset (A) and the Paris basic dataset (B): minimum (red line), mean (green line) and maximum (blue line).

The mean observed RGR (Figure 1) was higher in Paris (0.076 aphid/aphid/day (1/day)) than in Rennes (0.047 aphid/aphid/day (1/day)), as were its maximal values (0.45 and 0.29, respectively). The minimal value of RGR is of the same order in both regions (−0.5).

### b) The Parametric Model

We used the deterministic function for f(.):
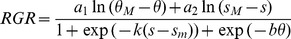
(4)


Where 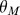 is the maximum lethal temperature, 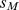 the latest wheat growth stage allowing aphid feeding, 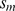 the position of the left inflexion point for the response to wheat growth stage. 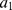,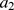, 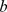 and 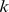 are parameters required to fit the model.

This function had the following features:

response to wheat growth stage is sigmoidal on the left, and falls sharply on the right until wheat reaches stage 92 (grain ripening);
response to the temperature increases until a thermal maximum (30°C), and then sharply decreases.

Equation (4) is an *ad hoc* response to these features well-known from experimental data [19], [31].

This model was therefore fitted to the observed RGR by non-linear ordinary least squares (*R, nls* function, Gauss-Newton method). After identification of the parameters (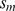, 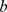, 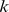, 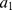 and 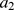), we used Equation (4) to predict the RGR, given temperature and wheat growth stage.

### c) The Predicted Aphid RGR and Temperatures

**Figure 2:**
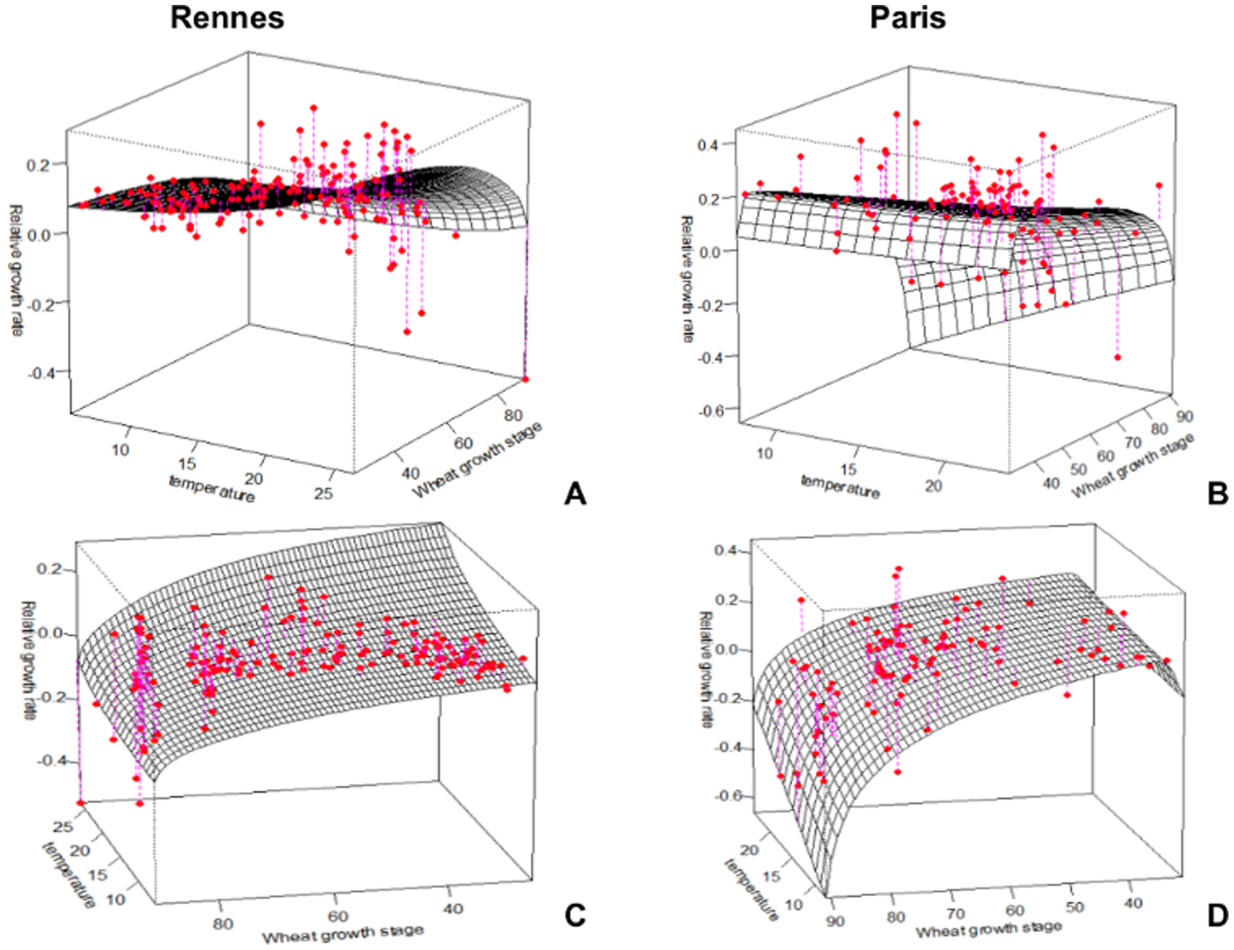
*S. avenae* Relative Growth Rate (RGR) according to temperature (A, B)) and wheat growth stage (C, D) for the Rennes and Paris basic datasets, respectively. Red circles, observed values; surface grid, modelled values.

For a given growth stage, aphid RGR increased progressively with temperature (Figures 2A and 2B). This was due to the direct effect of temperature on the intrinsic rate of increase in aphids [19].

### d) The Predicted Aphid RGR and Wheat Growth Stage

To analyse the relationship between aphid RGR and wheat growth stage, we divided Figures 2C and 2D into three parts:

- 1^st^ part: from stem elongation (stage 30) to heading (stage 50), there was a plateau in the modelled RGR values around 0.1 for the Rennes data, and an increase from 0.02 to a value around 0.15 at heading for the Paris data.
- 2^nd^ part: After the heading stage, aphid RGR decreased slowly to medium milk stage (stage 75) in both regions.
- 3^rd^ part: After stage 75, aphid RGR decreased sharply until grain ripening (stage 90) in both regions.

### e) Estimation of the Model Parameters from the Basic Datasets

We fit equation (4), setting 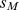 to 92, 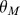 = 30 and 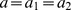. We obtained the following values for the coefficients:

- For the Rennes dataset: 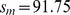, 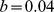, 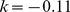 and 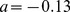;
- For the Paris dataset: 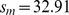, 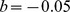, 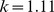 and 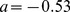.

All parameter correlations were lower than 0.95 in absolute value (Tables 1 and 2), except the correlation between *a* and *b* which appeared to be very strongly correlated. We were unable, however, to find a reparametrisation of Equation (4) that would reduce this correlation.

**Table 1:** Correlation of parameter estimates for the model based on the Rennes dataset.

	S_m_	b	k
b	0.52		
k	0.52	0.41	
a	0.53	0.99	0.33

**Table 2:** Correlation of parameter estimates for the model based on the Paris dataset.

	s_m_	b	k
b	−0.24		
k	−0.70	0.14	
a	−0.30	0.96	0.18

**Figure 3:**
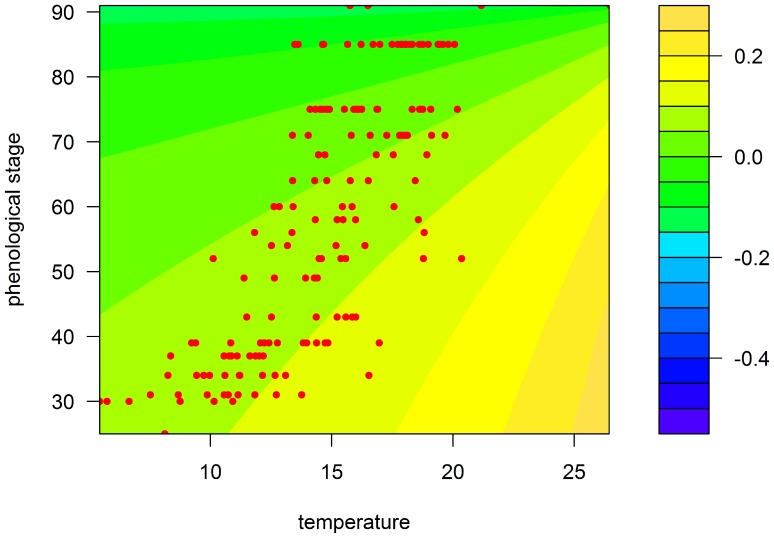
Prediction map of the model for the Rennes region according to temperature (°C) and wheat growth stage (Zadoks scale). Coloured areas, predicted RGR; red circles, observed RGR.

**Figure 4:**
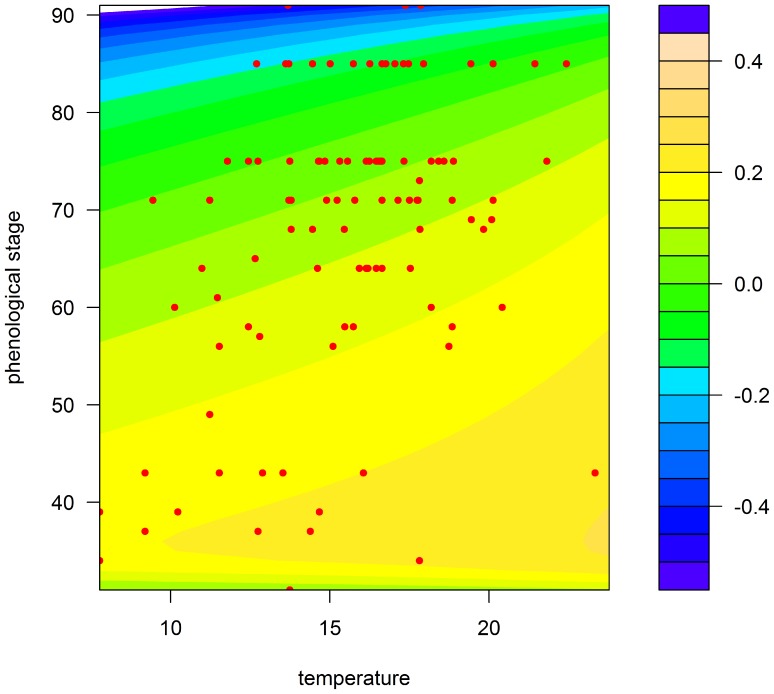
Prediction map of the model for the Paris region according to temperature (°C) and wheat growth stage (Zadoks scale). Coloured areas, predicted RGR; red circles, observed RGR.

Figures 3 and 4 show in three dimensions how the surface model fit the observed points. The value of R^2^ for our model was equal to 26.30% for Rennes and 50.81% for Paris. Although the model explains only a small proportion of the total variance, the analysis of variance (ANOVA) showed that this proportion was highly significant, with a *P*-value equal to 9.57e-10 (Table 3) for Rennes and 2.62e-12 (Table 4) for Paris. The Durbin-Watson test indicated a positive autocorrelation of residuals (DW = 0.83, *P*-value = 5.27e-14 for Rennes and DW = 1.09, *P*-value = 2.37e-06 for Paris). Close to 1, this positive autocorrelation was significant, but moderate. It indicates however that the *F* values may be inflated and the standard deviations of the coefficients underestimated. Nevertheless, the estimation of standard deviation is only asymptotic in the case of non-linear regression, and is not known very accurately.

**Table 3:** Analysis of variance for the Rennes-based model.

Source	SS	df	MS	*F*	*P*-value
Total	1.8189	159	0.011439		
Model	0.4783	4	0.119583	13.94	9.57335e-10
Error	1.3382	156	0.008578		

SS, sums of squares; df, degrees of freedom; MS, mean squares; *F, F* test.

**Table 4:** Analysis of variance for the Paris-based model.

Source	SS	df	MS	F	*P*-value
total	3.025	101	0.02995		
model	1.537	4	0.38430	20.52	2.62135e-12
error	1.835	98	0.01872		

SS, sums of squares; df, degrees of freedom; MS, mean squares*; F, F* test.

When a linear relationship with the weekly numbers of aphids killed by two natural enemies, Entomophthorale fungi, and parasitoids, is included in the model for the Rennes dataset, we obtain a new model:



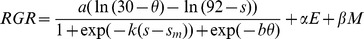
(5)
*E* stands for the number of aphids killed by Entomophthorale fungal disease, and *M* for the number of mummies (cadavers of aphids killed by a parasitoid wasp) in the sample.

*θ* represents temperature; *s*, wheat growth stage and coefficients had the following values: 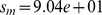, 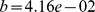, 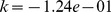, 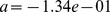, 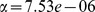 and 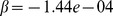.

The value of R^2^ is slightly better (27.70%) than in the model without natural enemies, with the density of aphids killed by natural enemies contributing significantly to the variance of the results. The Durbin-Watson test value was 0.83 and the *P*-value was 5.02e-14.

### f) Validation of the Model on the Test Dataset

**Figure 5:**
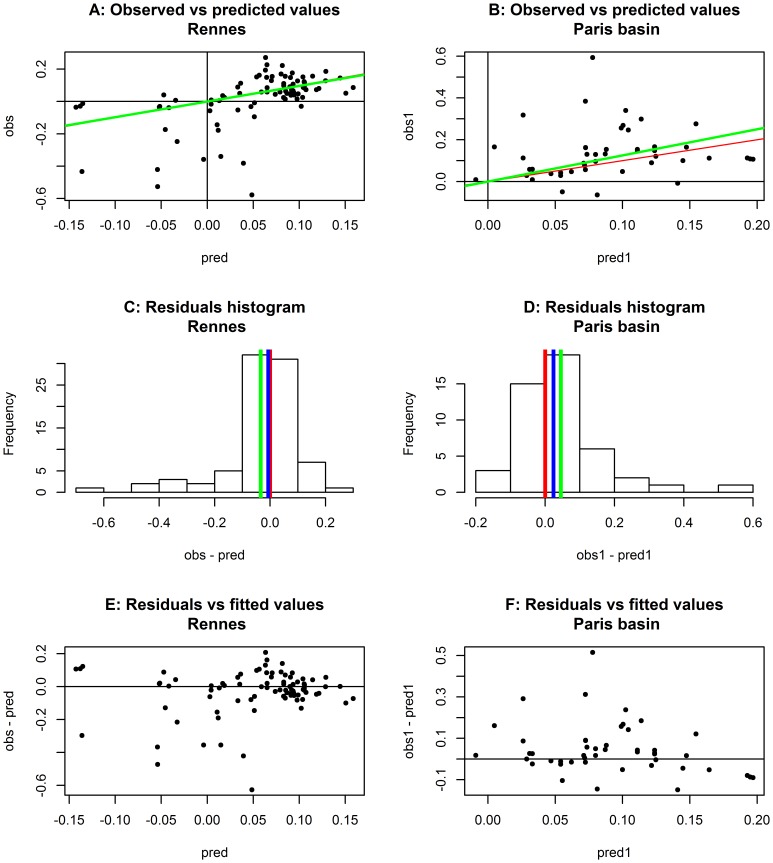
Validation of the models for the Rennes (left-hand panels, A, C, E) and Paris (right-hand panels, B, D, F) datasets without natural enemies. Plot of observed versus predicted values (A, B); red line, bisector (equality of prediction and observation); green line, regression line constrained to origin. Histograms of prediction residuals (C, D); red line, zero residual (desired mean); green line, mean of residuals; blue line, median. Plot of residuals against predicted values (E, F).

In the case of the Rennes and Paris test datasets, regarding overall quality of prediction (Equation 4), values of R^2^ were 24.90% and 49.60%, respectively. The predictive quality of the models was far better for the Paris dataset than for the Rennes dataset. Figures 5A and 5B show the plots of the predicted values against the observed values for the Paris and Rennes study fields, respectively. The figures show that there was little bias, with slopes close to approximating unity: t comparison between Paris (1.25), and Rennes (0.97), suggests that the prediction bias was very small. The R^2^ of these regressions was very close to that calculated for the bisector: R^2^ = 23.10% for Rennes and R^2^ = 49.50% for Paris.

Figures 5C and 5D show the histogram of the residuals in both cases that show residuals to be strongly skewed, positively for the Rennes dataset (Figure 5C) and negatively for the Paris dataset (Figure 5D). The similarity of the mean and the median to zero confirm the weak bias of the prediction average, but indicated some skewness in variability, particularly affecting large values of RGR observed for the Paris dataset, and small values in the Rennes dataset. This skewness is confirmed by the plot of the residuals against the predicted values (Figures 5F and 5E), for which there is no simple ecological explanation. Some possible reasons will be discussed below.

We examined the proportion of cases where RGR was predicted in the wrong direction (predicted to be positive, but where a negative value is observed and vice versa). In these cases, the movement of the population is growing when decreasing and decreasing when actually growing according to sign error matrices (Tables 5 and 6). However, there were relatively few observed cases: it was found that 14 cases (16.67% of the total) occurred in Rennes and only 4 cases (8.51% of the total) in Paris.

**Table 5:** Error matrix for the Rennes validation model.

	Predicted values	
	Positive	Negative
Observed values		
Positive	58	2
Negative	12	12

**Table 6:** Error matrix for the Paris validation model.

	Predicted values	
	Positive	negative
Observed values		
Positive	43	1
Negative	3	0

## Discussion

There was no single model with common parameters for the Rennes and Paris regions, probably due to the different climatic factors acting on the two populations. Any possible common model would have required accepting a very poor fit and even poorer validation. Aphid population dynamics, when observed in the field, varies greatly between an oceanic climate, as exemplified in Rennes, and a more continental one, such as experienced in the Paris region. The continental RGR was generally higher than the oceanic one, even with the same conditions of temperature and plant phenology (Figure 1). One explanation is that, in oceanic areas, overwintering parthenogenetic aphids spend winter with their natural enemies, which exert a continuous, although hardly detectable, deleterious pressure affecting their RGR. In contrast, more continental areas are free of aphids and their enemies at the end of winter because parthenogenetic aphid forms have been killed by very cold, freezing conditions. Continental areas are invaded in early spring by healthy, immigrating aphids that multiply without being regulated by natural enemies.

Despite the difficulties to estimate accurately aphid RGR from field data, we found a satisfying qualitative agreement between this estimation and the data obtained from the literature [19], [32].

For a given temperature, the estimated RGR was quantitatively and noticeably lower than that observed in laboratory experiments [19]. In laboratory experiments, there is no mortality factor other than temperature itself, but in the field, aphid mortality occurs randomly. Natural populations are subject to attacks from parasitoids, entomophthorales and predators (coccinellids, syrphids, chrysopids, carabids, spiders) that reduce the growth rate of aphids. The maximum predicted RGR was about 0.25 in both Rennes and Paris. These values were also lower than the maximum observed by Dean [19], which was about 0.3. Dean [19] found these maxima occurred at 22°C. The slope of increase between 6 and 22 degrees was steeper in Paris than in Rennes. Since there were no observed mean daily temperatures higher than 22°C in all our data records, we could not observe the phase of decrease in the RGR above 22.5°C, as described by Dean [19] and reported for many insects [33], [34].

The relationship of RGR with wheat growth stage is more difficult to compare with controlled experiments, because this type of study is scarce [31], due to the great difficulty of setting them up. However, we found what was expected: a plateau or an increase in the aphid RGR from early wheat growth stages to heading, followed by a decline. This feature was clearly observed, especially in the Paris region. From stem elongation to heading, we observed an increase in RGR linked to immigration and reproduction [35], [36]. From heading to ripening, the decrease in RGR was mainly due to a low intrinsic rate of increase and to emigration [1], both of which are responses to the decrease in the nutrient quality of the host [8], [37], as well as to the action of natural enemies [16], [31].

All these points of agreement with laboratory studies indicate that, although the random residuals around the model are large, the model extracts a signal from the data that is identifiable and coherent.

In contrast to expectations, temperature did not appear to be the most influential factor driving aphid RGR. The effect of the wheat growth stage appeared to be much more important. There are two possible explanations for this result:

Firstly, the effects of these two factors are strongly confounded in field observations, because both plant growth and aphid development depend heavily on temperature.
Second, the observation design also unavoidably confounds temperature and phenology. It is very unlikely to observe advanced plant growth stages at low temperatures because these stages occur in the summer and, likewise, to observe early growth stages at high temperatures because these stages occur in the early spring. This is clearly visible in Figures 3 and 4, where each pair of temperature and plant growth stage is confined to a delimited diagonal area in the plots.

In addition to these confounding effects, field data is also subject to complex environmental noise and sampling errors. Thus, focusing on only two model predictors may seem questionable because many other effects can influence aphid RGR. Two important natural enemy groups were included (Entomophthorale fungal diseases and parasitoid wasps) that increased model complexity, but did not help improve the model fit (R^2^, 27.7% vs. 26.3% without natural enemies). This may be attributed to two possibilities: (1) our observations are biased, reflecting the recent past rather than the actual effect of these antagonists; (2) we ignored the effect of many well-known polyphagous aphid enemies, such as coccinellids, syrphids, chrysopids, carabids and spiders. It would be worthwhile to include data on these polyphagous predators, but they are unlikely to become available for forecasting models [10], [16]. Furthermore, including this kind of data, even in detailed deterministic models, is difficult, because the occurrence of these predators is quite irregular and unpredictable. Although fundamentally interesting, multiple predator-prey dynamics involve an immense sampling effort that is impractical to carry out for the purposes of forecasting.

Results of the validation process show that the fitted models are much more predictive in the Paris region than in the Rennes region. Nevertheless, both exhibit a very weak bias on average, neither overestimating nor underestimating the RGR values, although the errors were strongly skewed in different directions in the two datasets. We obtained good qualitative validations. However, unexpectedly high values were often observed, and poorly predicted for the Paris region whereas in the Rennes region, unexpectedly low negative values were observed. These contrasted types of error may be due to the differences in climate, whereby, the mild oceanic climate in Rennes allows aphids to coexist throughout the year with their regulators, whereas the populations of the Paris region result mainly from immigrants from the west, joined later by the populations of parasites, predators and diseases. These events cannot be observed at a large scale easily, and are thus not taken into account in our non-linear models. However, the overall absence of bias indicates that host plant stage and the temperature are the main factors that explain the population dynamics of these insects. Our two models are therefore useful, with their estimated parameters, as a comprehensive population dynamics model.

Finally, fitting the effects of temperature and host plant phenology on observed data has seldom been done for insect populations. Even though models based on laboratory experiments may produce better predictions [38], our model is probably more robust and realistic because it is based on data collected in natural conditions. Furthermore, many combinations of temperature and phenology occur *in natura* that are almost impossible to mimic properly in an experimental design.

Lastly, our RGR modelling would be a convenient sub-model for large scale spatio-temporal forecasting. For example, in a reaction-diffusion system [39] representing spatially explicit dynamics of *S. avenae*, replacing a constant reaction term by our RGR model improve the model’s overall predictive and explanatory power [40]. Both such RGR and reaction-diffusion models were designed for practical use and for scientific research purposes. In practical terms, our objective is to develop a decision-support system underpinned by RGR models and field data collected from aerial trapping of aphids in a European network of suction traps [41], [42]. In terms of research, these models can be used to help predict the effect of global warming on the spread and increase of pest aphid populations [4]. Our models thus represent the first, essential albeit arduous, step to these future applications.
